# Welcome to pneumonia.org.au

**DOI:** 10.15172/pneu.2012.1/210

**Published:** 2012-05-10

**Authors:** Allan W. Cripps

**Affiliations:** Griffith University, Gold Coast Campus, Queensland, 4222 Australia

It is with great pleasure that I welcome you to **pneumonia**.

In 2006, I had the privilege to co-chair with Amanda Leach the Fifth International Symposium on Pneumococci and Pneumococcal Diseases (ISPPD). Prior to the meeting, an international panel led by Michael Alpers and Kim Mulholland assembled a discussion paper which called for a global action plan [GAP] against childhood pneumonia. This paper was discussed at ISPPD and with some modifications, it was accepted by the conference delegates to become known as the ISPPD declaration [1]. During the course of ISPPD5, many positive comments were received on the value of the international panel in advancing the fight against childhood mortality caused by pneumonia and suggestions were made for the development of a forum to raise the global profile of pneumonia as a significant disease in its own right. At the most recent ISPPD meeting held in Iguacu Falls, Brazil, in May of this year, Kim Mulholland led a session dedicated to briefing the ISPPD community on the advances that had been made in the roll out of the Global Action Plan for the Prevention and Control of Pneumonia (GAPP) as it has now become known. He appropriately titled his introduction “Historical overview of the GAPP. From Alice Springs to Iguacu Falls” — the session outlined significant advances that have been made over the last 6 years. However, despite these advances, pneumonia remains the leading cause of child death worldwide and is responsible for over two million deaths annually of children under 5 years of age. Nearly one in five of all child deaths are caused by pneumonia with most of them occurring in developing countries [2].

In 2009, I co-chaired a tri-nation meeting on childhood pneumonia with Bob Douglas in Sydney. This meeting was attended by 43 delegates from Indonesia, Papua New Guinea, Australia and from international donor agencies to discuss further local initiatives aimed at reducing child mortality and morbidity from pneumonia, as well as support in the Asia-Pacific region for the GAPP initiative [3]. At this meeting, the discussion groups again raised the possibility of a regular forum in which information specific to pneumonia could be disseminated.

In comparison to other childhood killers such as malaria and diarrhoea, the situation for pneumonia is much more complicated. The diagnosis is not straight forward, the decision to treat with antibiotics is often ambiguous and preventative interventions are complicated, in that, a multipronged approach is necessary. Risk factors associated with the environment and pollution need to be addressed. What is required is a preventative approach based on effective case management practices, vaccination and strategies to improve childhood nutrition and correct deficiencies in essential micronutrients such as zinc. Hence, as recognised by the World Health Organisation (WHO) in the mid 1990’s when the concept of the Integrated Management of Childhood Illness program was introduced, the prevention of pneumonia requires a child specific rather than a disease specific approach [4]. As a consequence and as recognised by the delegates who attended ISPPD5 and the Tri-nation meeting, information on pneumonia is distributed across a plethora of journals and publication avenues.

**pneumonia** is a peer reviewed, online, open access journal. It is dedicated to establishing an international forum for pneumonia in the broadest context and as a means for bringing together knowledge related to pathogenesis, treatment and prevention of pneumonia. The journal will publish original research articles, case studies, reviews, critical commentaries, correspondence, highlights and news on all aspects of pneumonia.

**pneumonia** will be published by Griffith University ePress. There are no fees for the submission of manuscripts, nor any costs associated with their publication or access online.

I am confident that over the coming years **pneumonia** will become a journal of choice for those wishing to publish high quality manuscripts in the field and establish a forum for discussion.

I invite you to visit **pneumonia** at pneumonia.org.au and to submit material for publication in the journal.
